# Internet Interventions or Patient Education Web sites?

**DOI:** 10.2196/jmir.8.3.e18

**Published:** 2006-09-29

**Authors:** Lee M Ritterband, Frances Thorndike

**Affiliations:** ^1^University of Virginia Health SystemCharlottesvilleVAUSA

In the paper “Internet Interventions for Long-Term Conditions: Patient and Caregiver Quality Criteria,” [1] the authors raise a timely concern with Internet interventions – consumers’ perspectives on the quality of these interventions. Given that consumers will ultimately decide the fate of Internet interventions, it is critical that we, as Internet intervention developers and researchers, solicit their thoughts and perspective in creating these programs. However, we have two major concerns that we believe limit what can be drawn from this paper.

The authors attempt to put forward “quality criteria” for Internet interventions based on 10 focus groups with a total of 40 participants. They were given time to examine the sites and then provide feedback. However, it appears that the list of “Internet interventions” were mostly patient education web sites and not what most in the field of Internet intervention research would consider Internet interventions [2-5]. Internet interventions are typically behaviorally or cognitive-behaviorally-based treatments that have been operationalized and transformed for delivery via the Internet. Usually, they are highly structured; self or semi-self guided; based on effective face-to-face interventions; personalized to the user; interactive; enhanced by graphics, animations, audio, and possibly video; and tailored to provide follow-up and feedback [2].

Perhaps it is the term “Internet intervention” that is problematic, as one could regard an interactive patient education web site to be an intervention. “Internet intervention” could be considered an umbrella term that encompasses various types of web programs, including behaviorally-based and empirically validated web-based treatment programs as well as patient education sites. However, currently, it is our belief that the term “Internet intervention” is not typically used as a generic phrase, but as the specific expression for what is described above. At present, the lack of formal terms to define these various web sites and web programs may be confusing to those not familiar with this area. However, with the significant use of the Internet for health purposes [6], it is important that standard terms be created and used to reduce confusion and to avoid the current practice of using these terms interchangeably.

Interestingly, in the introduction to the paper, the authors describe and cite Internet intervention research that fits the more specific definition above. In fact, two of the authors wrote a paper for the Cochrane review in 2005 evaluating this literature [7]. Near the end of the introduction, the authors even state that “A further limitation of most quality criteria and previous user perspective research is that they do not distinguish between sites which contain information only and interactive sites which combine information with decision support, behavior change support, or peer support.” The authors clearly set the stage to investigate such web-based programs (Internet interventions) but then fail to apply these criteria to the selection process of the interactive health communication applications (IHCAs) used in the current paper. In fact, only a couple of the web sites used in the focus groups for this paper even seem to come close to the criteria of what is typically considered true Internet interventions (i.e., the CHESS programs by David Gustafson’s group). Basically, the “results” in this paper are drawn from patient education sites (i.e., heartcenteronline.com and alzheimersdisease.com – and many more), and not from Internet interventions as they are described above. To conclude their investigation by saying that participants “…felt that many [Internet interventions] were not achieving their full potential” is misleading given that most participants did not actually view or use true Internet interventions. It is also not surprising that participants believed these sites were not achieving their potential as patient education sites typically provide only a small component of what a true Internet intervention usually offers.

In this paper, the authors also inquired about and reported what “good Internet interventions” should contain or be (i.e., “A good Internet intervention will provide information about the following…”). These global statements are also flawed and misleading. First, they are again based on participants’ viewing of patient education web sites (and not real Internet interventions). Second, it is unclear how the authors determined which criterion were important. Was there a threshold for determining that a criterion was worthy of follow-up examination in the respondent validation survey (e.g., 50% of participants mentioned it)? Third, it is difficult, and sometimes meaningless, to try to generalize across interventions/disorders. Interventions can (and should) be significantly different from program to program given the focused disorder. For example, we would not expect a program targeting diabetes to contain the same ingredients as a program targeting insomnia. Fourth, to say “a good Internet intervention will provide information about ‘medication’ or ‘available treatments in the UK and elsewhere’” is much too general a statement to be made. Instead, offering some of the bulleted items as issues for consideration would be more appropriate. Some useful, though-provoking information was obtained by the focus group members, and researchers, clinicians, and developers in the field of Internet interventions could learn from this contribution. However, the provision of the information as currently presented is at times misleading and the conclusions drawn are inaccurate.

This paper does raise the important point that there is a need for an authoritative body to provide information about, and possibly rate the quality of, Internet interventions. This should include not only patient and caregiver criteria, but empirically validated outcome studies demonstrating effectiveness. We hope the current paper and subsequent discussion will help provide the impetus to push this critical agenda forward. Similarly, there is an obvious need to better define what Internet interventions are and how to differentiate among various kinds of health-focused web sites. As we move forward in this young field, we must clarify how we communicate about these interventions to ensure productive exchanges and sound science behind our work.
